# Is there a correlation between prepartum anaemia and an increased likelihood of developing postpartum depression? A prospective observational study

**DOI:** 10.1007/s00404-023-07344-7

**Published:** 2024-02-12

**Authors:** Marco La Verde, Mario Luciano, Mario Fordellone, Carlotta Brandi, Marco Carbone, Matteo Di Vincenzo, Davide Lettieri, Marica Palma, Maria Maddalena Marrapodi, Gaetano Scalzone, Marco Torella

**Affiliations:** 1 Department of Woman, Child and General and Specialized Surgery, Obstetrics and Gynecology Unit, University of Campania “Luigi Vanvitelli”, Naples, Italy; 2 Department of Psychiatry, University of Campania, “L. Vanvitelli”, Naples, Italy; 3 Medical Statistics Unit, University of Campania Luigi Vanvitelli, Naples, Italy; 4 Department of Woman, Child and General and Specialized Surgery, Pediatric Unit, University of Campania Luigi Vanvitelli, Naples, Italy

**Keywords:** Pregnancy, Perinatal depression, Edinburgh Postpartum Depression Scale, Postpartum depression, Haemoglobin, Materna anaemia

## Abstract

**Purpose:**

Postpartum depression (PPD) represents a significant challenge to maternal and child health. Early screening for PPD is essential to ensure appropriate treatment and support. The present study aimed to assess whether maternal prepartum anaemia influences the likelihood of developing PPD within 3 days after delivery.

**Methods:**

In collaboration with the Department of Psychiatry, a prospective observational study was carried out at the Gynaecology and Obstetrics Department of the University of Campania “Luigi Vanvitelli” in Naples. A total of 211 full-term pregnant women were enrolled, and their predelivery haemoglobin value was recorded. Women with gestational diabetes, hypertension, pre-eclampsia, intrauterine growth restriction, intellectual disability, or pre-existing diagnosis of psychotic spectrum disorder were excluded. Participants provided written informed consent to fill out the Edinburgh Postnatal Depression Scale (EPDS) 3 days after delivery. EPDS cut-off score of ≥ 10 was used to identify women at risk of developing PPD. Statistical analysis was performed using Student's t test, the Wilcoxon Rank Sum test, and linear regression.

**Results:**

The participants were categorized into 2 groups based on EPDS scores: EPDS < 10 (176 patients) or EPDS ≥ 10 (35 patients). The two groups showed homogeneity in terms of socio-demographic and clinical characteristics. The mean haemoglobin values of anaemic pregnant women in the EPDS ≤ 10 group (11.78 ± 1.39 g/dl) and the EPDS > 10 group (11.62 ± 1.27 g/dl) were not significantly different (*p* = 0.52). There was no significant correlation between the predelivery haemoglobin value and the EPDS postpartum score of < 10 or ≥ 10. The Wilcoxon Rank Sum test and the estimated coefficients of the linear regression model did not show any statistical relationship between continuous and binary haemoglobin values.

**Conclusions:**

Our study found that maternal prepartum anaemia did not negatively impact the likelihood of developing postpartum depressive symptoms, in the first 3 days after delivery.

## What does this study adds to clinical work

**Table:** 

This study contributes to current knowledge by providing evidence that prepartum anaemia does not increase the likelihood of developing postpartum depression within three days of delivery, nor does it influence the anhedonia, anxiety, and depression subscales of the Edinburgh Postnatal Depression Scale. Our findings contribute to clinical practice by establishing a basis for future investigations into the potential correlation between pregnancy anaemia and postpartum depression.	

## Introduction

Anaemia is one of the most significant global public health issues impacting people's physical and mental abilities [1]. Geography, lifestyle, and diet influence the prevalence of anaemia in pregnant women, estimated to range between 14 and 80% in various countries [2–5]. Recently, consideration has been given to the function of iron deficiency anaemia (IDA). Women who have had IDA during pregnancy are more likely to have a delayed recovery of iron reserves in the postpartum period. The prevalence of IDA during pregnancy is around 7.5% [6, 7]. Behavioural symptoms associated with anaemia in adults include changes in cognition, emotions, irritability, apathy, fatigue, depressive symptoms, and hypoactivity [7, 8]. Even though anaemia during pregnancy is more prevalent in countries with low or middle incomes than in countries with high incomes, the prevalence of anaemia in these countries is still high, especially in Asia [9]. In Europe, the prevalence of IDA is 21–35% [10]. Several studies have demonstrated that anaemia plays a role in the development of adverse pregnancy outcomes, such as pre-eclampsia, premature rupture of membranes, low birth weight, preterm birth, and fetal and maternal mortality [11–13]. Because anaemia may produce weariness, irritability, lethargy, and depressive symptoms during pregnancy or the postpartum period has been identified as a possible physiological risk factor for PPD (PPD) [5, 9, 14]. The potential mechanisms by which anaemia causes depressive symptoms are generally explained by altering myelinisation and neurotransmitter metabolism [15].

PPD typically occurs between 6 and 12 weeks postpartum [16, 17]. Depending on the diagnostic criteria and screening instruments used, the prevalence of PPD ranges between 10 and 15% [18]. In addition to affecting the mother’s mental health, it disrupts family relationships and the child’s emotional and cognitive development [19–25]. PPD is a major risk factor for suicide among postpartum mothers worldwide [26, 27], requiring a pharmacological intervention [25] apart from psychological interventions usually provided to women with PPD [28]. Several psychological risk factors have been identified in PPD, including a previous history of depression, elevated levels of postnatal stress, and inadequate social and financial support during the postpartum period [29–33]. However, limited research has been conducted on physiological variables that may contribute to the development of PPD [34] and currently, it is not known whether this disorder can be considered a clinical entity psychopathologically different from Major Depressive Disorder [35, 36]. The Edinburgh Postnatal Depression Scale (EPDS) is a widely regarded screening tool for detecting postpartum depression [37], despite other instruments are available [38]. The EPDS effectively identifies, with good sensitivity and specificity, women susceptible to developing PPD [39]. In the 1980s, John Cox, Jeni Holden, and Ruth Sagovsky developed the EPDS in Edinburgh, Scotland [40]. The authors aimed to develop a tool capable of detecting symptoms of depression and identifying the severe and persistent ones that require clinical intervention [40]. The EPDS is typically administered on the third day after delivery and repeated at subsequent follow-up appointments [41]. The mother self-administered the questionnaire, which should take less than 5 min to complete [42]. EPDS consists of 10 items that assess the presence and severity of symptoms of depression. The items are scored on a four-point scale ranging from 0 to 3, with scores ranging from 0 to 30 [43]. The EPDS total score is calculated by summing the scores of each item, with higher scores indicating greater severity of depressive symptoms [44]. The recommended cut-off points for the EPDS vary depending on the study design and population being examined [45]. A score of 13 or above is suggested to indicate severe depressive symptoms, but a score of 10 or above is commonly used to screen for PPD [46]. The sensitivity and specificity of different cut-off points vary, and local prevalence rates and available resources should inform the choice of cut-off point [45, 47]. However, it is essential to note that the EPDS is a screening tool, not a diagnostic instrument [48]. A healthcare professional should further evaluate any woman who scores positively on the EPDS, and clinical judgment is necessary to determine the presence and severity of postnatal depression [49]. In this prospective study, we used a validated depression score (EPDS and EPDS subscales) to explore the occurrence of prepartum anaemia and its potential correlation with PPD. Timely screening for postnatal depression is essential to ensure appropriate treatment and support for affected women. Identifying and managing PPD promptly is crucial in order to prevent adverse outcomes. Early recognition of a risk factor such as prepartum anaemia is crucial for promoting the mental health of both mothers and their children.

## Materials and methods

The present study was a longitudinal observational study conducted at the Gynaecology and Obstetrics Department of the University of Campania “Luigi Vanvitelli” in Naples, in collaboration with the Department of Psychiatry. All full-term pregnant admitted to our institution’s delivery room between December 2019 and February 2021 were invited to participate. We excluded women with a severe intellectual disability or a pre-existing diagnosis of schizophrenia, schizoaffective disorder, delusional disorder, bipolar or other unspecified psychotic spectrum disorder. Furthermore, in order to achieve a homogeneous obstetric population and avoid bias, we excluded pregnancies complicated by gestational and pregestational diabetes, chronic hypertension, gestational hypertension, pre-eclampsia/eclampsia, intrauterine fetal growth restriction, preterm delivery, multiple pregnancies and pregnancies with fetal abnormalities detected prenatally. The participants were enrolled before the onset of labor and provided their written informed consent after understanding the study’s objectives. Following the delivery, precisely 3 days postpartum, participants were instructed to fill out the EPDS Italian version [50]. Upon enrollment, the following information was collected for all pregnant women: (1) socio-demographic characteristics (age, level of education, marital and employment status); (2) clinical information on pregnancy in progress (spontaneous previous abortions, vaginal delivery vs caesarean, presence of obstetric complications during pregnancy including eclampsia and gestational diabetes, the Apgar index at the 1st and 5 min); (4) social and contextual factors (relationship with the partner, family conflicts, socio-economic situation); (5) all data regarding the blood chemistry tests performed at the time of admission. All patients carry out the usual routine blood chemistry tests provided by our facility at hospitalization in accordance with good medical practices. The haemoglobin value obtained from the hospitalisation tests was utilised as an indicator for evaluating the existence of maternal anaemia. As per the definition provided by the World Health Organization (WHO), the diagnosis of anaemia in the third trimester of pregnancy is established when the concentration of haemoglobin falls below 11.0 g/dL [51]. As previously mentioned, we used the EPDS on the third day after delivery to evaluate depressive symptoms in the postpartum period. The EPDS is a validated questionnaire developed to detect postnatal depression. The questionnaire consists of ten self-reported items rated on a four-point scale. The EPDS has been found to have good sensitivity and specificity, making it effective in identifying postnatal depression onset. Various cut-off points have been determined in validation studies [45]. A score of 13 or above is suggested to indicate severe depressive symptoms, but a score of 10 or above is commonly used for screening postnatal depression [46]. In this study, we used a cut-off ≥ 10 to identify women at increased risk of developing PPD [37]. Three subscales were extracted from the EPDS [52], namely the anhedonia subscale (items 1 and 2), the anxiety subscale (items 3 to 6) and the depression subscale (item 7 to 10). This study was conducted following globally accepted standards of good clinical practice, in accordance with the Declaration of Helsinki and with national and local regulations. The study protocol was approved by the Ethics Committee of the University of Campania “Luigi Vanvitelli” (protocol number 98 of February 28, 2019).

### Statistical analysis

Continuous variables were reported as either the means and standard deviation or median and interquartile ranges (IQRs) according to their distribution, as assessed by the Shapiro–Wilk normality test. Categorical variables were reported as percentages. Differences in characteristics of patients between EPDS groups were tested by *t*-test or Wilcoxon rank sum test (according to their distribution) and Pearson chi-squared or Fisher's exact test (according to their distribution) for continuous and categorical variables, respectively. To measure the linear association between continuous variables, Pearson correlation test was used if variables had a normal distribution, otherwise Spearman’s rank correlation test was calculated.

To measure the effect of the HGB on the EPDS, four regression models were used. In particular, two linear regression model were estimated using the EPDS scale as continuous response variable and HGB as predictor variable in continuous and binary form, respectively; moreover, two logistic regression model were estimated using the categorized EPDS as binary response variable and the same predictors (i.e., continuous and categorized HGB variable). All the regression coefficients were adjusted for several socio-demographic, clinical and contextual baseline patients’ characteristics.

All the statistical analyses were performed by the R Studio Statistical software, version 4.1.3.

## Results

**Fig. 1:**
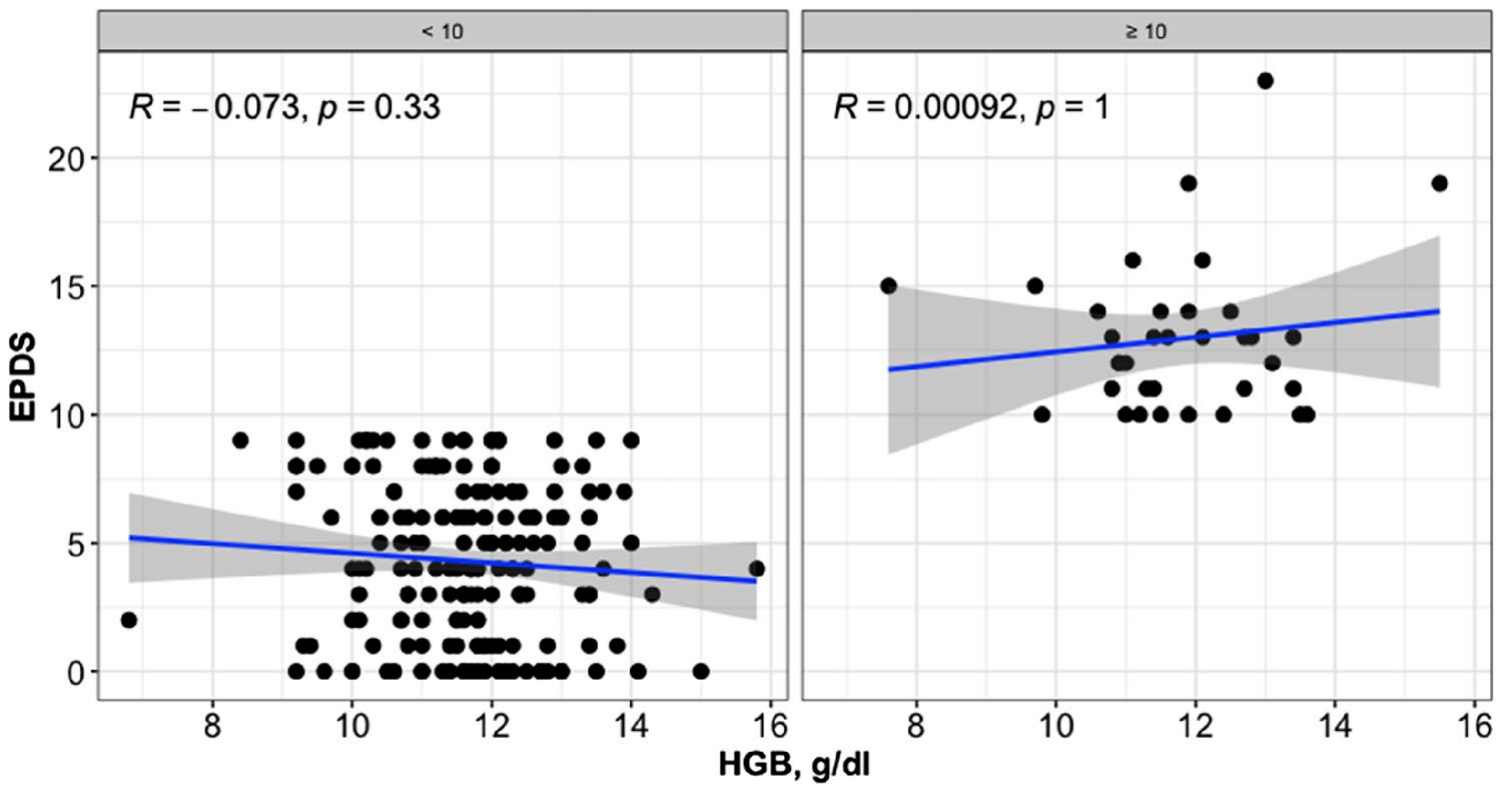
Scatter-plot with the Spearman’s rank correlation test on HGB level and EPDS scale stratified for EPDS groups

**Fig. 2:**
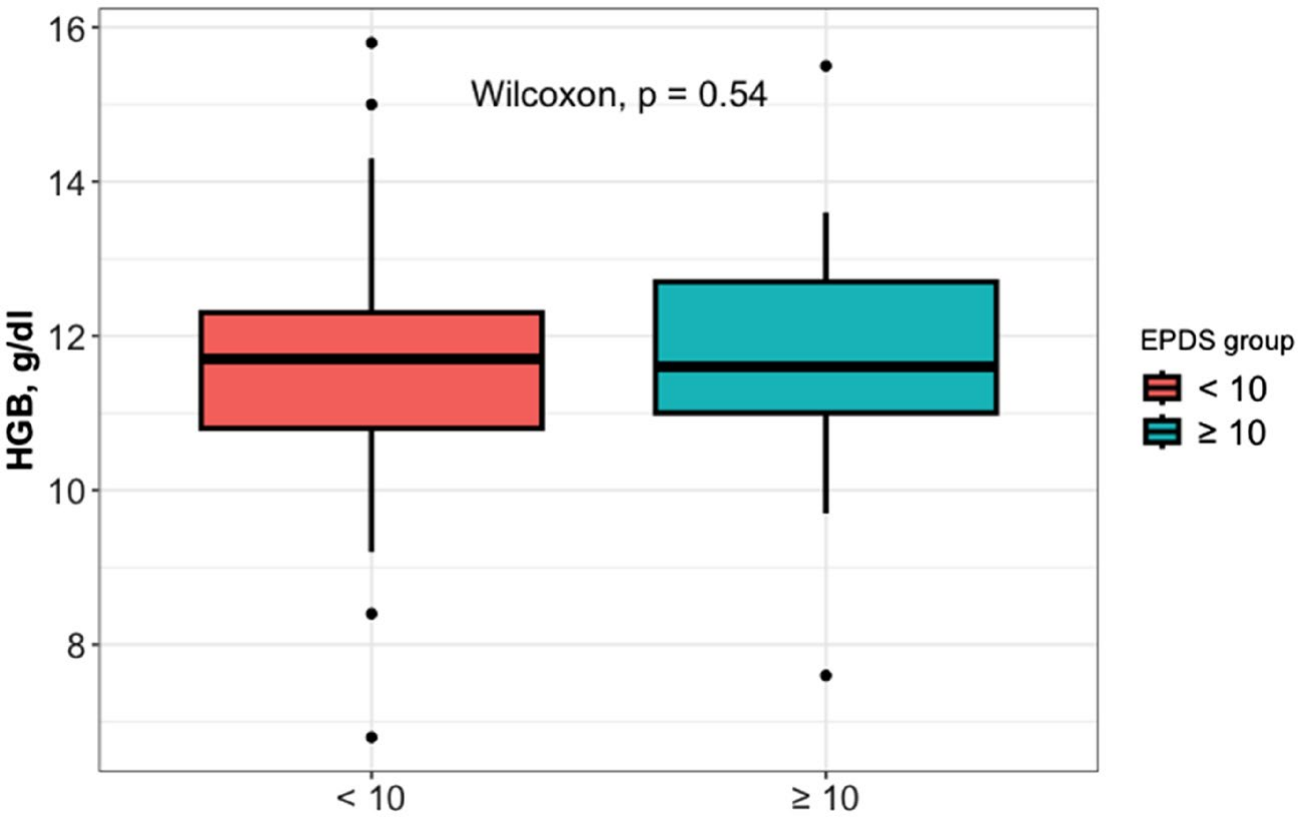
Box-plot with the Wilcoxon rank sum test on HGB level and EPDS groups

A total of 234 pregnant women met the inclusion criteria. All participants were invited to take part, however, only 211 individuals agreed to participate. All enrolled patients’ haemoglobin (Hb) values were obtained from the routine admission venous blood sample. The participants were categorised into 2 groups based on the EPDS ≤ 10 (176 patients) or > 10 (35 patients). Based on the Hb status, EPDS ≤ 10 group included 8 anaemic pregnant versus 47 anaemic pregnant of EPDS > 10 group (mean Hb values of 11.78 ± 1.39 g/dl versus 11.62 ± 1.27 g/dl, p value 0.52). Hence, it can be observed that the two groups show homogeneity in terms of age, education, prior BMI, and gestational age at delivery, employment and Apgar score, as no statistical differences were found when comparing these variables (Table 1). The women enrolled in the study had a median age of 32.00 (± 7.75) years. Additionally, a significant proportion of these women (40.6%) possessed a good level of education (Table 1). No statistical distinctions were observed between the two groups regarding cigarette consumption patterns (8.5% were smokers—Table 1). No significant differences were observed in the clinical information regarding current pregnancy, as well as the social and contextual aspects, between the two groups. These factors included the (relationship with the partner, family conflicts and socio-economic situation (Table 1). A Scatter-plot of the Hb level and EPDS scale stratified for EPDS groups is shown in Fig. 1. A postpartum EPDS score of ≤ 10 or > 10 did not correlate significantly with the predelivery Hgb value (Table 2). We have also employed the Wilcoxon Rank Sum test on Hb levels and EPDS groups to investigate the haemoglobin levels of the pregnant women in the two study groups. Figure 2 presents the box plot comparison of the two data sets. The Wilcoxon Rank Sum test did not indicate a statistical relationship. The estimated coefficients of the linear regression model with continuous and binary HGB values indicated the absence of a statistical relationship (Tables 3, 4 and 5). The sub-analysis of the EPDS subscales also did not show significant statistical differences between pregnant with or without anaemia (Table 6).

**Table 1:** Socio-demographic and clinical characteristics of the sample

Characteristic	Overall, *N* = 211^1^	EPDS		*p* value^3^
		< 10, *N* = 176^2^	≥ 10, *N* = 35^2^	
Age	32.00 (8.00)	32.00 (8.00)	32.50 (6.50)	1.00
Prior BMI	23.51 (4.41)	23.56 (4.03)	22.45 (5.64)	0.34
Maternal ethnicity				0.89
White	198.0 (93.8%)	164.0 (93.2%)	34 (97.1%)	
Hispanic	7.0 (3.3%)	6.0 (3.4%)	1 (2.9%)	
Asian/Pacific Islander	6.0 (2.8%)	6.0 (3.4%)	0 (0.0%)	
Smoke				0.52
No	193.0 (91.5%)	162.0 (91.8%)	31.0 (88.6%)	
Yes	18.0 (8.5%)	14.0 (8.2%)	4.0 (11.4%)	
Marital status				
Single	58.0 (27.5%)	47.0 (26.7%)	11.0 (31.4%)	0.69
Married	152.0 (72.0%)	117.0 (66.5%)	24.0 (68.6%)	
Divorced	1.0 (0.5%)	1.0 (0.6%)	0.0 (0.0%)	
Pregnancy weeks				0.56
37	9.0 (4.3%)	9.0 (5.1%)	0.0 (0.0%)	
38	26.0 (12.3%)	20.0 (11.4%)	6.0 (17.1%)	
39	74.0 (35.1%)	60.0 (34.1%)	14.0 (40.0%)	
40	66.0 (31.3%)	57.0 (32.4%)	9.0 (25.7%)	
41	36.0 (17.1%)	30.0 (17.0%)	6.0 (17.1%)	
School				0.87
Bachelor’s or equivalent level	17.0 (8.1%)	14.0 (8.0%)	3.0 (8.6%)	
Lower secondary education	58.0 (27.5%)	47.0 (26.7%)	11.0 (31.4%)	
Master’s or equivalent level	44.0 (20.8%)	37.0 (21.0%)	7.0 (20.0%)	
Upper secondary education	92.0 (43.6%)	78.0 (44.3%)	14.0 (40.0%)	
Employment				0.14
No	95.0 (45.0%)	75.0 (42.6%)	20.0 (57.1%)	
Yes	116.0 (55.0%)	101.0 (57.4%)	15.0 (42.9%)	
Previous abortions	58.0 (27.5%)	48.0 (27.3%)	10.0 (28.6%)	0.84
Conflicts with partner	19.0 (9.0%)	15.0 (8.5%)	4.0 (11.4%)	0.53
Financial problems	12.0 (5.7%)	8.0 (4.5%)	4.0 (11.4%)	0.06
Having a partner with depression	6.0 (2.8%)	5.0 (2.8%)	1.0 (2.9%)	1.00
Having a partner with an anxiety disorder	12.0 (5.7%)	8.0 (4.5%)	4.00 (11.4%)	0.06
Mode of conception				0.89
Spontaneous	205.0 (97.2%)	170.0 (96.6%)	35.0 (100%)	
IVF	6.0 (2.8%)	6.0 (3.4%)	0.0 (0.0%)	
Apgar 5 min	9.00 (1.00)	9.00 (1.00)	9.00 (1.00)	0.60
Apgar 1 min	8.00 (1.00)	8.00 (1.00)	8.00 (1.00)	0.07

^1^Median (IQR); *n* (%); ^2^Median (IQR) or frequency (%);^3^Wilcoxon rank sum test; Fisher’s exact test

**Table 2:** Estimated coefficients of the linear regression model with continuous HGB

Characteristic	Beta	95% CI	*p* value
HGB	0.025	− 0.528, 0.577	0.93
Age	0.002	− 0.121, 0.125	0.97
Prior BMI	− 0.024	− 0.175, 0.127	0.76
Smoke			
No	–	–	
Yes	− 0.149	− 2.557, 2.258	0.90
Pregnancy weeks	0.013	− 0.610, 0.637	0.97
School			
Bachelor’s or equivalent level	–	–	
Lower secondary education	− 0.395	− 3.174, 2.384	0.78
Master’s or equivalent level	− 1.941	− 4.834, 0.952	0.19
Upper secondary education	− 0.528	− 3.219, 2.164	0.70
Employment			
No	–	–	
Yes	− 1.040	− 2.512, 0.432	0.17
Apgar 1 min	0.498	− 0.305, 1.302	0.22
Apgar 5 min	0.180	− 1.123, 1.484	0.78

Estimates adjusted for baseline patients characteristics

*CI* confidence interval

**Table 3:** Estimated coefficients of the linear regression model with binary HGB

Characteristic	Beta	95% CI	*p* value
HGB group			
< 11	–	–	
≥ 11	0.033	− 1.524, 1.589	0.97
Age	0.002	− 0.121, 0.126	0.97
Prior BMI	− 0.023	− 0.175, 0.128	0.76
Smoke			
No	–	–	
Yes	− 0.143	− 2.556, 2.270	0.91
Pregnancy weeks	0.012	− 0.611, 0.636	0.97
School			
Bachelor's or equivalent level	–	–	
Lower secondary education	− 0.395	− 3.176, 2.386	0.78
Master's or equivalent level	− 1.938	− 4.842, 0.966	0.19
Upper secondary education	− 0.526	− 3.217, 2.165	0.70
Employment			
No	–	–	
Yes	− 1.043	− 2.513, 0.427	0.16
Apgar 1 min	0.493	− 0.301, 1.288	0.22
Apgar 5 min	0.186	− 1.110, 1.482	0.78

Estimates adjusted for baseline patients characteristics

*CI* confidence interval

**Table 4:** Estimated odds ratio of the logistic regression model with continuous HGB

Characteristic	OR	95% CI	*p* value
HGB	1.236	0.888, 1.737	0.21
Age	0.988	0.917, 1.064	0.76
Prior BMI	0.983	0.890, 1.072	0.72
Smoke			
No	–	–	
Yes	1.054	0.222, 3.751	0.94
Pregnancy weeks	0.972	0.666, 1.426	0.88
School			
Bachelor’s or equivalent level	–	–	
Lower secondary education	1.454	0.303, 10.732	0.67
Master’s or equivalent level	0.767	0.133, 6.156	0.78
Upper secondary education	0.874	0.185, 6.373	0.88
Employment			
No	–	–	
Yes	0.806	0.337, 1.936	0.63
Apgar 1 min	1.838	1.003, 3.787	0.075
Apgar 5 min	0.811	0.322, 1.999	0.65

Estimates adjusted for baseline patients characteristics

*OR* odds ratio, *CI* confidence interval

**Table 5:** Estimated odds ratio of the logistic regression model with binary HGB

Characteristic	OR	95% CI	*p* value
HGB group			
< 11	–	–	
≥ 11	1.761	0.670, 5.318	0.28
Age	0.991	0.920, 1.067	0.81
Prior BMI	0.984	0.891, 1.073	0.73
Smoke			
No	–	–	
Yes	1.126	0.237, 4.042	0.87
Pregnancy weeks	0.981	0.673, 1.437	0.92
School			
Bachelor’s or equivalent level	–	–	
Lower secondary education	1.431	0.296, 10.634	0.68
Master’s or equivalent level	0.742	0.126, 6.031	0.75
Upper secondary education	0.888	0.186, 6.523	0.89
Employment			
No	–	–	
Yes	0.786	0.330, 1.881	0.58
Apgar 1 min	1.833	0.988, 3.808	0.081
Apgar 5 min	0.828	0.326, 2.055	0.69

Estimates adjusted for baseline patients characteristics

*OR* odds ratio, *CI* confidence interval

**Table 6:** EPDS and EPDS subscale

	Anaemia^1^ (*n* = 55)	Control Group^1^ (156)	*p* value
EPDS *n*°(%)	8 (14.5)^2^	27 (17.3)^2^	0.83^2^
Anhedonia subscale, Mean ± SD	0.78 ± 0.84	0.61 ± 0.85	0.25
Anxiety Mean ± SD	3.69 ± 2.79	5.80 ± 2.40	0.46
Depression Mean ± SD	1.67 ± 1.93	1.37 ± 1.83	0.31

Abbreviation: *EPDS* Edinburgh Postnatal Depression Scale

^1^t-Test; ^2^Fisher’s exact test

## Discussion

The scientific community is debating different risk factors associated with an increased likelihood of developing PPD. The precise identification of risk factors associated with the development of PPD prior to delivery could significantly support physicians in managing this commonly underestimated condition, and to tailor treatments for women with depressive symptoms during the postpartum period [53], also il line with women treatments preferences [54]. The relationship between anaemia during the third trimester of pregnancy and PPD remains unclear due to conflicting results in various studies. Additionally, no research has focused on the connection between maternal anaemia and the subscales of the EPDS. Our research revealed no link between prepartum anaemia and an increased probability of developing symptoms of PPD within three days after delivery. Furthermore, our analysis determined that the EPDS subscales did not correlate with prepartum anaemia. A recent meta-analysis of 15 studies examines the connection between anaemia and the risk of maternal depression [55]. The findings demonstrate a significant association between anaemia and an increased risk of maternal depression, with an odds ratio of 1.53 [55]. However, of the included studies, only seven assessed postpartum symptoms. Notably, Albacar et al. evaluated depressive symptoms and obtained blood samples 48 h after delivery, focusing only on iron storage levels and the inflammatory marker [56]. Alharbi et al. [57], through an observational case–control and retrospective study, revealed a correlation between pregnancy anaemia and EPDS after 8–12 weeks postpartum in Saudi Arabia. Chandrasekaran et al. specifically examined the relationship between PPD and postpartum anaemia only in women who underwent elective caesarean sections [58]. Eckerdal et al. investigated the impact of heavy postpartum haemorrhage on PPD onset [59]. Another research conducted by Azita Goshtasebi et al. explored the influence of prepartum haemoglobin levels on PPD among urban pregnant women in Iran [60]. This study suggests that a haemoglobin level below 11 g/dL at delivery may increase the likelihood of experiencing PPD (EPDS cut-off adopted > 13). It should be noted that this finding is specific to haemoglobin levels and not necessarily related to iron deficiency. The study sample included healthy women at low risk for complications, and none of the women in this study had iron deficiency anaemia at delivery (low haemoglobin and ferritin values) [60]. Surkan et al. [61] assessed PPD in a low-income country using their own scale, adapted from the Patient Health Questionnaire (PHQ-9) and the Center for Epidemiologic Studies Depression Scale (CES-D). Xu et al. [62] examined hospitalization rates for anaemia and depression in women three years before and three years after birth. It is crucial to highlight that the studies mentioned in the meta-analysis are heterogeneous and differ in terms of scales, EPDS cut-off points, and timing for postpartum EPDS administration when examining the correlation between PPD and maternal anaemia [55]. In another recent study, Maeda et al. administered EPDS four weeks after the delivery and the blood test was performed in the second and third trimesters and one week after the delivery. The authors, with an EPDS cut-off of 9, demonstrated how low Hb levels in the first week of the postpartum period were significantly associated with an increased PPD risk, while no such correlation was observed between anaemia or Hb levels during the second and third trimesters and PPD risk [9]. Corwin et al. analyzed 37 women and reported how the low haemoglobin level seven days after delivery was negatively correlated with self-reported depressive symptoms at day 28 [6]. Corwin et al. adopted the Center for Epidemiological Studies-Depressive Symptomatology Scale (CES-D) to screen for depressive symptoms.

The precise connection between prepartum anaemia and PPD is not completely clear, and one possibility is that postpartum anaemia causes behavioural and psychological symptoms similar to those induced by iron deficiency anaemia: it is a form of anaemia caused by iron deficiency, and its mechanism differs from that of second and third-trimester anaemia, which may occur due to a physiological increase in plasma volume (hemodilution) [9]. Our findings, for the first time, demonstrated how prepartum anaemia does not modify the likelihood of developing PPD in the immediate postpartum period. Simultaneously, we observed that anaemia did not influence the EPDS subscale, which includes the anhedonia, anxiety, and depression subscales. The study possesses several notable strengths. Firstly, it adopts a prospective design with a prospective collection of data. Additionally, the study benefits from a large population. Moreover, the data collection adopted a scientifically validated assessment tool, the EPDS, administered within three days after the delivery. It is worth noting that the EPDS screening for PPD, and the immediate postpartum period were not previously correlated to peripartum anaemia. Moreover, to attain a uniform obstetric population, we specifically recruited individuals with uncomplicated pregnancies, excluding those with gestational and pregestational pathologies: gestational and pregestational diabetes, chronic hypertension, gestational hypertension, pre-eclampsia/eclampsia, intrauterine fetal growth restriction, preterm delivery, multiple pregnancies and pregnancies with fetal abnormalities detected prenatally. At least the diagnosis of anaemia was performed homogeneously and systematically in our hospital laboratory. However, it is essential to acknowledge the limitations of our study. Firstly, there may be methodological limitations in our estimation of PPD prevalence as we just relied on the EPDS screening tool to assess depressive symptoms. A definitive diagnosis of PPD can only be made through a structured psychiatric interview, which we offered to all patients who tested positive for EPDS. Second, due to the exclusive collection of haemoglobin data during hospitalization, the correlation between anaemia during each trimester of pregnancy and the EPDS results was unavailable. Third, we have not collected data on iron, vitamin B12, folic acid levels, and other factors that cause maternal anaemia. Nevertheless, additional research is required to elucidate the potential implications of anaemia on the subsequent postpartum period (after three days of delivery). Furthermore, clinical trials are necessary to assess the efficacy of anaemia treatment during pregnancy in mitigating the risk of PPD.

## Conclusion

According to our study's findings, maternal prepartum anaemia did not negatively impact the likelihood of developing postpartum depressive symptoms, as measured by the EPDS, in the first three days following the delivery.

## Data Availability

The data that support the findings of this study are not publicly available due to privacy reasons but are available from the corresponding author upon reasonable request.
